# The Use of Glenoid Structural Allografts for Glenoid Bone Defects in Reverse Shoulder Arthroplasty

**DOI:** 10.3390/jcm13072008

**Published:** 2024-03-29

**Authors:** Helen Ingoe, Kristine Italia, Luke Gilliland, Hean Wu Kang, Mirek Karel, Jashint Maharaj, Kenneth Cutbush, Ashish Gupta

**Affiliations:** 1 Queensland Unit for Advanced Shoulder Research (QUASR), Queensland University of Technology, Brisbane, QLD 4000, Australia; helen.ingoe@otago.ac.nz (H.I.); kristine@akunah.com (K.I.); luke@akunah.com (L.G.); heanwu@hotmail.com (H.W.K.); mirekkarel@outlook.com (M.K.); jashint@qoc.com.au (J.M.); k.cutbush@uq.edu.au (K.C.); 2 Akunah, Brisbane, QLD 4120, Australia; 3 School of Medicine, University of Queensland, Brisbane, QLD 4072, Australia; 4 Queensland Orthopaedic Clinic, Greenslopes Private Hospital, Brisbane, QLD 4000, Australia

**Keywords:** autograft, allograft, reverse shoulder arthroplasty, union, glenoid, BIO-RSA

## Abstract

**Background:** The use of reverse shoulder arthroplasty as a primary and revision implant is increasing. Advances in implant design and preoperative surgical planning allow the management of complex glenoid defects. As the demand for treating severe bone loss increases, custom allograft composites are needed to match the premorbid anatomy. Baseplate composite structural allografts are used in patients with eccentric and centric defects to restore the glenoid joint line. Preserving bone stock is important in younger patients where a revision surgery is expected. The aim of this article is to present the assessment, planning, and indications of femoral head allografting for bony defects of the glenoid. **Methods:** The preoperative surgical planning and the surgical technique to execute the plan with a baseplate composite graft are detailed. The preliminary clinical and radiological results of 29 shoulders which have undergone this graft planning and surgical technique are discussed. Clinical outcomes included visual analogue score of pain (VAS), American Shoulder and Elbow Surgeons score (ASES), Constant–Murley score (CS), satisfaction before and after operation, and active range of motion. Radiological outcomes included graft healing and presence of osteolysis or loosening. **Results:** The use of composite grafts in this series has shown excellent clinical outcomes, with an overall graft complication rate in complex bone loss cases of 8%. **Conclusion:** Femoral head structural allografting is a valid and viable surgical option for glenoid bone defects in reverse shoulder arthroplasty.

## 1. Introduction

Glenoid wear and/or glenoid defects are commonly encountered during reverse shoulder arthroplasty (RSA). Different options for addressing glenoid defects are available depending on the pattern and severity. Minimal wear and minor deformity can be addressed by eccentric reaming. However, the use of this technique is limited as it is only effective for less than 15-degree glenoid deformity. More severe glenoid defects can be addressed by bony reconstruction or metallic augments. Bony reconstruction of the glenoid entails the use of structural bone grafts. Many surgeons generally prefer autografts from the humeral head as it is readily available in primary surgery. However, there may be certain situations in which the use of an autograft is not feasible. This may be in the case of revision surgery, fracture, or in the presence of previous rotator cuff repair, avascular necrosis, or osteoporosis, where the bone in the humeral head may be insufficient or mechanically weak. Other autograft options include iliac bone grafting; however, this has the issue of donor site morbidity that should not be understated and is often difficult to harvest in a beach chair position, especially in the obese [1]. 

A femoral head allograft is the usual allograft of choice for most surgeons as it is often readily available from bone banks. As allografts are inspected and treated before use, there is little risk of poor graft quality or risk of infection [2]. There are wider concerns by many that bony increased offset-reverse shoulder arthroplasty (BIO-RSA), whether by auto- or allograft, has low union rates and consequently results in implant failure [3,4]. Hence, some surgeons prefer using metallic augments or custom glenoids in these cases.

This article focuses on the use of structural allografts for glenoid bone defects in RSA. The clinical rationale and options for glenoid allografting will be discussed. Outcomes and complications after RSA with structural allografts for glenoid grafting will also be presented.

## 2. Glenoid Anatomy, Wear, and Defects

### 2.1. Types of Glenoid Bone Defects

**Figure 1:**
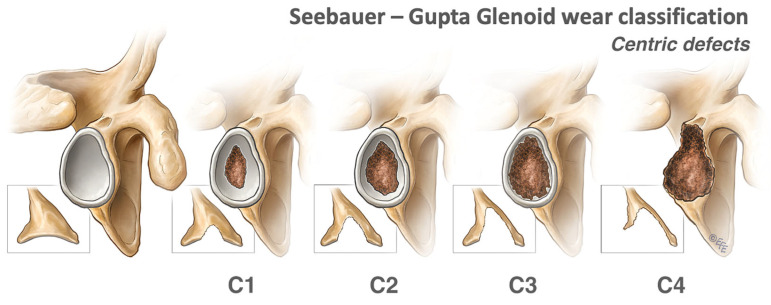
Centric defects according to the Seebauer–Gupta Classification [7]. C1 defects involve less than 50% of the anteroposterior (AP) glenoid diameter, while C2 defects involve more than 50% of the AP glenoid diameter with stable vaults. C3 defects are cavitary defects with unstable vaults. C4 defects have significant glenoid and vault destruction. (This figure was reproduced with permission from *Journal of Shoulder and Elbow Surgery*, Volume 27, Gupta et al., “Management of glenoid bone defects with reverse shoulder arthroplasty: surgical technique and clinical outcomes”, pages 854–862, copyright Elsevier (2018)).

**Figure 2:**
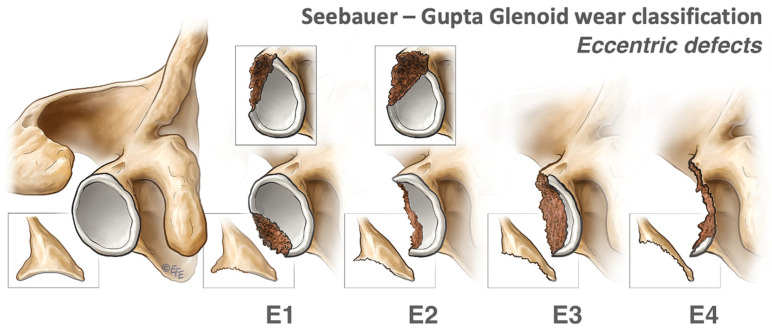
Eccentric defects, according to the Seebauer–Gupta Classification [7]. E1 defects are small or shallow eccentric defects. E2 defects involve less than 30% of the glenoid bone stock, while E3 defects involve between 30 to 60% of the glenoid bone stock. E4 defects are massive defects involving more than 60% of the glenoid bone stock. (This figure was reproduced with permission from *Journal of Shoulder and Elbow Surgery*, Volume 27, Gupta et al., “Management of glenoid bone defects with reverse shoulder arthroplasty: surgical technique and clinical outcomes”, pages 854–862, copyright Elsevier (2018)).

The standard glenoid wear pattern in osteoarthritis has been described by Walch [5,6]. However, this classification is insufficient when describing bone loss in more complex cases such as fracture or revision surgery. Glenoid bone defects in these cases are described by the Seebauer-Gupta classification and are grouped into concentric and eccentric defects (Figure 1 and Figure 2) [7]. The purpose of defining the defect is to guide treatment planning with small, contained defects often treated with eccentric reaming and large uncontained defects to be treated with structural composite grafting techniques. For successful implant integration and longevity, the glenoid baseplate graft composite is required to meet all the three parameters of the 50% rule: a minimum of 50% of the length of the long central peg in the native scapula, at least two opposite locking screws (superior and inferior) in the native scapula, a minimum of 50% of the baseplate-bone graft composite resting on the native glenoid [7]. When all parameters are met, the stability of the construct is ensured, even in cases of severe glenoid defects. This also dictates the feasibility of doing a single-stage procedure in revision cases wherein varying degrees of glenoid bone defects are usually expected.

#### 2.1.1. Centric Defects

Centric glenoid bone defects are usually the consequence of advanced glenoid wear but can occur secondary to polyethylene-induced aseptic loosening or rocking horse phenomena requiring revision surgery. Centric defects can also be encountered during revision surgeries after explantation of the baseplate, which can leave large cavitary defects. Centric defects are graded C1 to 4, with higher grades indicating greater depth of the defect (Figure 1). Grades C1 to C3 have an intact peripheral ring of bone, which is still structurally sound [7]. They are contained and have a stable vault and, therefore, can be treated with reaming and non-structural impaction bone grafting as required. Progressive C3 and C4 defects have an unstable vault and thus require a structural graft. 

#### 2.1.2. Eccentric Defects

Eccentric defects can be the cause of advanced wear and aseptic loosening but are more apparent secondary to instability, trauma, and notching. Similarly, eccentric defects are classified E1 to 4 depending on the percentage of glenoid bone stock loss (Figure 2), with E1 and E2 amenable to eccentric reaming [7]. E3 and E4 require structural grafting, which can reconstruct part of the joint surface or, in more advanced cases, can reconstruct the vault.

### 2.2. Indications for Glenoid Grafting

The indications for glenoid grafting are manifold and can include glenoid wear or trauma [8], bony defects following revision surgery [9], or as a method of lateralization [8]. The decision to use a bone graft will depend on the quantity and shape of bone loss, the need for implant longevity, and the anticipated need for revision. Other methods of tackling bony wear and defects include eccentric reaming [10] and metal augments [11], which may be suitable in older patients where a revision surgery is not anticipated and the need for future bone stock is not a priority.

### 2.3. Joint Line Restoration

The presence of glenoid erosion usually causes medialization of the glenoid articulating surface, subsequently medializing the joint line as well. This negatively impacts soft tissue tension and joint reaction forces [12]. Hence, it is the technical aim of many surgeons to restore the premorbid version and glenoid joint line. Benefits include increased stability of implants, restoration of soft tissue tension, and greater range of postoperative movement [13]. It is known that the normal glenoid joint line is, on average, 28.36 mm from the scapular notch and 11.66 mm from the centre of the coracoid [12]. The average medialization from the paleoglenoid rim to the neoglenoid base of a B2 glenoid measured on a three-dimensional (3D) computed tomography (CT) scan is, on average, 4 mm [14]. There can be errors in point-to-point measurement whilst quantifying 3D defects; thus, when a similar measurement is made using a statistical shape model (SSM) glenoid, a B2 glenoid is classified as an E2C2 defect, and often, there is a joint line medialization of around 1.2 cm at the deepest points.

#### 2.3.1. Lateralization

**Figure 3:**
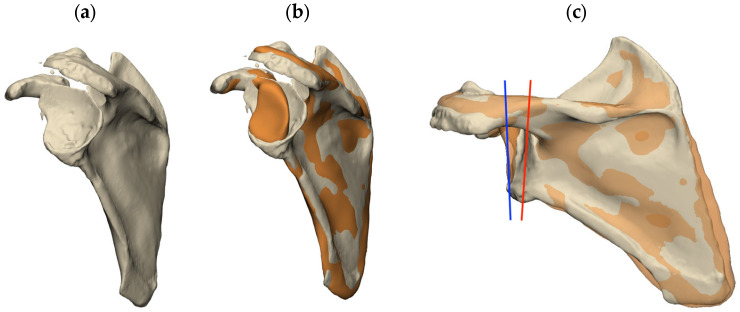
The use of SSM to predict the premorbid glenoid anatomy. (**a**) 3D model of the pathological glenoid. (**b**) Premorbid glenoid, as predicted by SSM (orange), overlayed with the pathological glenoid. (**c**) This can provide information on the amount of medialization in the pathological glenoid (red line) and the reconstruction necessary to restore the native joint line (blue line). (Image from Akunah, Brisbane, Australia).

Lateralizing beyond the native joint line can reduce scapular notching, improve impingement-free range of motion, and increase the deltoid wrap effect [8]. However, over-lateralization can lead to increased stress on the acromion that can result in fracture, inability to repair the subscapularis muscle, or stretching on the brachial plexus and conjoint tendon, causing plexopathy and pain [15]. The amount of lateralization to which the risks outweigh the benefits will differ in each patient and is yet to be accurately defined. However, to avoid excessive lateralization, the joint’s normal ‘goal line’ must first be known, and preoperative 3D CT-based planning comparing the unaffected contralateral shoulder should be employed. In cases where the contralateral shoulder is also pathological or when imaging is not available, the use of SSM can be employed to predict the premorbid glenoid, allowing the surgeon to assess the amount of glenoid bone loss and the size of the graft necessary to restore the normal joint line and avoid over-lateralization (Figure 3). On average, a 12 mm graft (either concentric, wedge, or “Figure 7”) produced 3 mm of lateralization past the normal joint line in a case series of 21 patients [16].

#### 2.3.2. Version and Inclination

For all diagnoses in the Australian Orthopaedic Association National Joint Registry Report 2023, the most common wear pattern for RSA was A1, followed by A2 [17]. B1 was then more observed in cuff tear arthropathy and fracture, while B2 was predominant in osteoarthritis. Conversely, C was the least frequently encountered pattern across all diagnoses [17]. In eccentric wear patterns, retroversion and superior inclination are the most commonly observed deformities [5,18]. The ideal glenoid version and inclination for maximum stability, range of motion, and impingement reduction are not universal. Patient characteristics and surgical goals can influence this choice. For example, in a younger patient with high functional expectations, an increased impingement-free range of motion may be targeted, whereas in a low functional demand patient, stability may be the most important surgical goal. The importance of internal and external rotational activities for individual patients may also influence version choice. Whatever the surgical goal, knowing the native glenoid version and inclination will assist in planning the optimum position [2,12,19].

Minor version and inclination deficits can be corrected with eccentric reaming at the expense of joint line medialization and potential baseplate stability and fixation [20]. Using an allograft to restore inclination and version allows the natural joint line to be restored or lateralized and creates a larger surface area to fit the glenoid baseplate [21]. 

## 3. Allograft Types

Both structural and non-structural allografts are used in the treatment of glenoid bone loss dependent on defect pattern and size. Allografts can be fresh-frozen or freeze-dried and can be harvested from multiple sites such as the femoral head and shaft, humeral head, and tibia.

### 3.1. Non-Structural Allografts

Freeze-dried bone is often used as a non-structural graft for bone impaction in contained defects. It is the least allogenic type of allograft and has the least structural integrity. Freeze-dried bone has the advantage of being almost universally readily available as it can be stored at room temperature and requires no preparation. This type of graft is used in contained defects such as C1, C2, and C3, which have the potential for impaction bone grafting, and standard flat back glenoids. The addition of platelet-derived growth factor is thought to help ingrowth of osteoblasts, but this has not been fully proven [22].

### 3.2. Structural Allografts

Structural allografts are fresh-frozen and can be harvested from live or post-mortem donors. They retain their mechanical properties but do not have osteoinductive or osteogenic properties [2]. They generally require pre-ordering from a bone bank and require defrosting perioperatively. Sterilization is performed by irradiation; however, this does not protect the donor from the transmission of HIV, which is identified through screening of the donor [2]. 

Bone grafts are usually ordered as whole bone (e.g., a femoral head) that can be shaped by freehand intraoperatively by the surgeon. If preoperatively planned, the size, shape, and orientation of the graft required to fill the defect can be preoperatively prepared by the bone bank or intra-operatively prepared using 3D-printed jigs to cut the exact shape required [23,24]. Typical bone graft shapes are concentric discs, wedges, and “Figure 7” grafts. The graft, in combination with a long-pegged baseplate, is known as a composite graft technique and is the favoured technique of the authors [7]. Alternatively, centric bone defects are filled by bone grafts and can be secured with the peg of the baseplate or with screw fixation [25].

#### Composite Grafts

**Figure 4:**
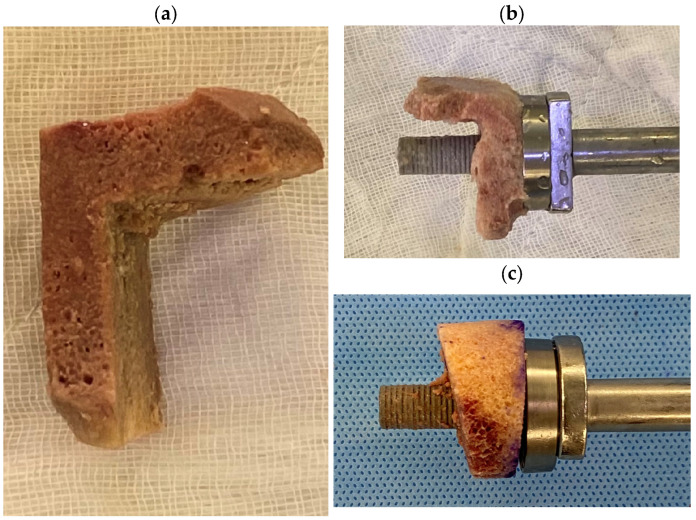
(**a**) “Figure 7” graft. (**b**) Composite graft wherein the “Figure 7” graft is combined with a long-pegged baseplate. (**c**) Composite wedge graft with long-pegged baseplate.

Concentric composite cylindrical grafts are ideal for restoring centric glenoid wear and increasing lateralization of the joint line (Figure 4). Wedge composite grafts are required in larger eccentric defects and are the most commonly used composite grafts in primary wear cases. “Figure 7” grafts are usually used for large eccentric defects secondary to trauma or chronic dislocation or in revision settings where part of the glenoid vault is compromised. Of note is that the minimum depth of the graft needed is 6–7 mm, as any graft that is thinner at its minimum depth may crack upon insertion of the baseplate. 

### 3.3. Bone Types

The most commonly used allograft is the femoral head, as it is abundantly available from bone banks and is of adequate size and structural integrity. Other donor sites include femoral shaft [26], humeral head, and tibia [27], with mixed results. In one study series, six out of seven femoral shaft grafts and three out of four proximal humeral grafts required revision surgery and removal of the grafts. This was for multiple different failure mechanisms in complex patients. However, as the rate of failure in the femoral head allograft group in the same series with the same surgeon was lower, it is likely that the type of graft is a factor [26].

### 3.4. Allograft Preparation

Allografts must be processed because of concern over microbial contamination and to reduce the risk of possible immunologic and inflammatory reactions in the recipient [28]. The most common techniques are putting the bone graft through an antibiotic wash and cooling it at −70 °C (fresh-frozen allograft) and the addition of lyophilization and irradiation (freeze-dried allograft) [29]. The hesitancy of some surgeons to use allografts stems from these, as these processes alter the biological properties of the bone grafts, which could affect their healing potential. There is still limited clinical data on this, with some studies favouring the use of fresh-frozen allografts [30] while others show comparable rates of union between the two [29].

### 3.5. Contraindications to Bone Grafting

Several contraindications exist for humeral autograft harvest that might indicate the need for allografting (Table 1). The presence of infection or tumor is an absolute contraindication. In patients where humeral autograft may be mechanically weak or insufficient, such as those with osteoporosis or avascular necrosis, this is a relative contraindication depending on the amount of bone affected. Implanting allografts in active infection is controversial; however, there are many successful individual cases of allograft implantation as part of single-stage revision [9]. The senior author has experienced very few glenoid grafting (either allo- or autograft) failures in their practice. A few cases of aseptic baseplate loosening of autografts have been observed to be associated with the use of denosumab perioperatively. Although no proven link has been established or published between this RANK Ligand monoclonal antibody and grafting failures, the authors advise caution against using denosumab during RSA due to this experience.

## 4. Preoperative Planning and Surgical Technique

Preoperative planning is imperative when performing RSA, particularly in complex cases and revision surgeries. This allows for a detailed evaluation of the pathological anatomy, which gives the surgeon information on the severity of the deformity and allows accurate planning for the surgery.

### 4.1. Preoperative Planning Technique

Patients’ CT scans are used to perform 3D preoperative planning of the glenoid with the implant and graft. Reflect^TM^ (Akunah, Brisbane, Australia) is used for preoperative planning. Scapular landmarks from the International Society of Biomechanics (ISB), including the Glenoid Centre (GC), Trigonum Spinae (TS), and Angulus Inferior (AI), are used [31]. Glenoid anatomical measurements such as version and inclination are identified.

**Figure 5:**
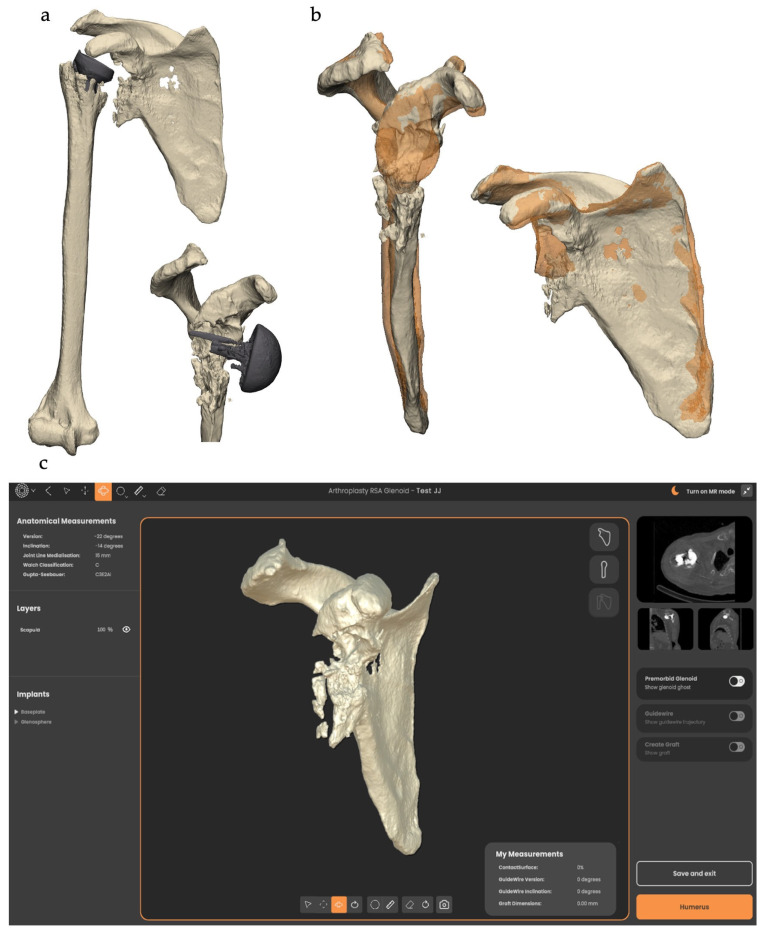
(**a**) 3D reconstruction of the pathological glenohumeral joint and failed implants. (**b**) Contralateral (orange) scapula overlayed onto the pathological scapula to assess glenoid bone loss and normal joint line. (**c**) Screenshot of Akunah Reflect^TM^ preoperative planning.

Glenoid wear and subsequent bone loss are assessed to provide information on implant and graft choice and positioning (Figure 5). Quantifying glenoid bone loss is performed in one of two ways: the mirroring and overlaying of the patient’s normal contralateral scapula onto the pathological, or the generation of an algorithm-based premorbid prediction via SSM in Reflect^TM^. Both methods provide valuable information about the severity and location of the bone loss to advise on the shape of the graft required to fill the defect. The thickness of the graft is also determined from these methods, as they have the ability to define the premorbid joint line, at which the glenoid lateralization/medialization is zero.

After examining the glenoid anatomy in 3D, analysing glenoid measurements, and assessing the severity of the bone loss, implants and grafts are chosen. The glenoid baseplate is positioned on the glenoid in such a way that takes all the provided information into account. Following this, the graft is generated as a projection from the medial surface of the baseplate to the glenoid surface. 

### 4.2. Surgical Technique

A standard deltopectoral approach is performed, followed by subscapularis peel and humeral head cut as needed. Care is taken to identify and protect the axillary nerve as it courses from under the subscapularis muscle under the glenoid and exits between teres minor and major. Complete 360-degree visualization of the glenoid is of paramount importance before performing any complex grafting manoeuvres. This is achieved by complete excision of the superior, middle, and inferior glenohumeral ligaments and scar tissue, if present, in conjunction with a 360-degree capsulectomy, to expose the complete face of the glenoid. Exposure of the lateral pillar and the medial 3 cm of the glenoid neck anteriorly and inferiorly with a complete release of the long head of triceps from its insertion is also performed. Then, based on the preoperative planning, the glenoid defect is classified as per the Seebauer–Gupta classification [7].

#### 4.2.1. Glenoid Preparation

The glenoid guidewire is inserted using a freehand technique or with guiding tools such as navigation, patient-specific instrumentation (PSI), or mixed reality. These recent advances in technology assist surgeons in reproducing the preplanned guidewire insertion and increase the accuracy of glenoid guidewire insertion. Once the guidewire is positioned, the glenoid cortical surface is inspected and prepared. Glenoid bed preparation is of paramount importance to ensure that there is backside bleeding from the cancellous bone to maximise graft incorporation. Extreme caution is advised when using a standard mechanized off-the-shelf circular reamer. In the senior author’s experience, the sclerotic bone of the glenoid is often very dense, and the rotatory motion of the standard reamers can lead to an iatrogenic glenoid fracture, especially in the setting of dense eburnated-like bone. 

**Figure 6:**
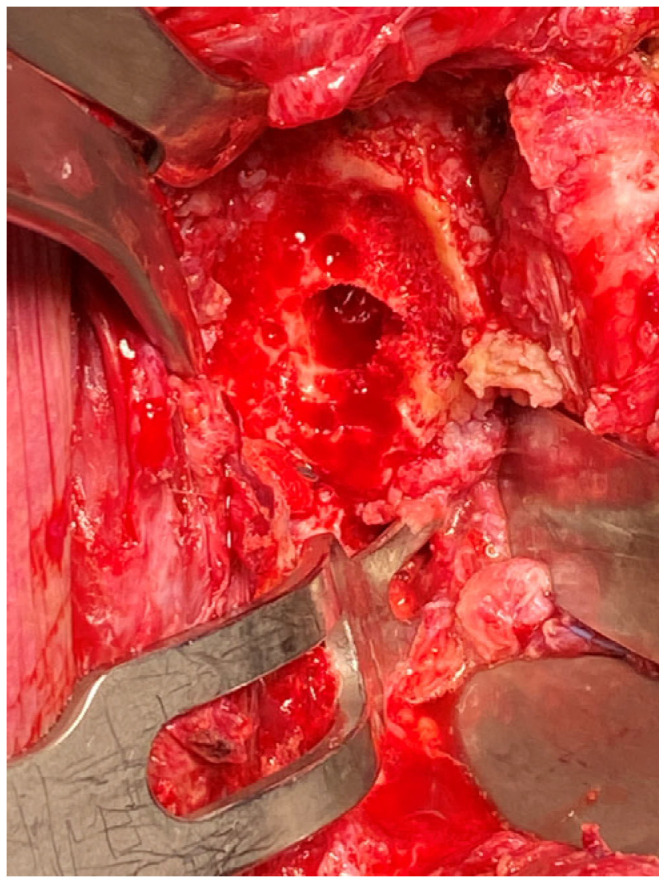
Prepared bed with a high-speed burr, demonstrating glenoid bed preparation with clean cancellous bleeding surface.

The senior author’s preference is to use a pineapple-shaped conical high-speed burr, and care is taken to smoothen the surface of the cortex of the en-face glenoid (Figure 6). In opposition to anatomic total shoulder arthroplasty, where the subchondral bone is not breached, in the setting of a complex glenoid allograft, the subchondral surfaces are breached to ensure sufficient bleeding from the cancellous bed. All osteophytes are then carefully removed using a bone nibbler. Care is taken to make radial cuts on the glenoid edges to avoid any propagation of an iatrogenic glenoid rim fracture. Once sufficient back bleeding is attained from the face of the glenoid, a complete glenoid preparation is then ascertained. 

#### 4.2.2. Baseplate 

A choice is made whether to use a central screw-based device with the allograft complex, or a peg-based glenoid baseplate. Based upon the 50% rule, as proposed in a previous publication [7], unless there is a contraindication, the senior author’s preference is to use a peg-based design.

#### 4.2.3. Preparation of the Structural Allograft

The defect is classified as a centric or an eccentric defect, or a combination thereof. The centric part of the defect can be filled with a mix of a cancellous and cortical impaction bone graft with a fresh-frozen allograft as a preference for ease of use.

In a combined centric and eccentric defect, a custom allograft can either be preoperatively planned and ordered from the bone bank to match the preoperative deformity based on the preoperative planning, or an on-table shape matching can be performed. 

##### Cylindrical and Wedge Grafts

**Figure 7:**
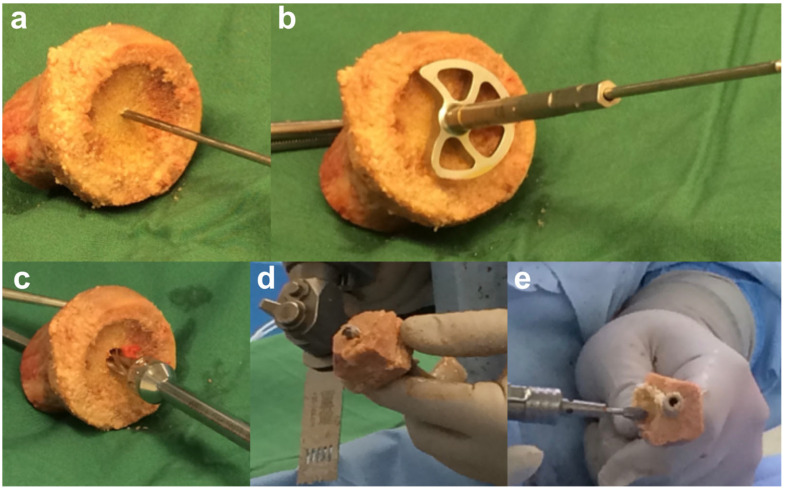
Preparation of the structural allograft. (**a**) K-wire is inserted into the femoral head, (**b**) reamed using circular reamer, (**c**) followed by the peg drill. (**d**) The femoral head is cut to appropriate size using a handheld saw and (**e**) shaped using a handheld high-speed burr.

A femoral head is usually used and preferred for a standard cylindrical graft or a 10° to 15° wedge graft. Following appropriate thawing, a Kirschner wire (K-wire) is inserted into the femoral head (Figure 7a). The entry point is then reamed using the circular reamer for the baseplate preparation (Figure 7b), followed by the peg drill (Figure 7c). The peg drill is left in situ, and the femoral allograft is cut to the appropriate size using a handheld saw (Figure 7d) and shaped using a high-speed burr (Figure 7e), usually allowing for a 25 to 29 mm diameter with varying depths of thickness (6–7 mm minimum) depending on the size of the defect and pre-planned lateralization. 

The graft is then loaded onto the backside of the glenoid baseplate, and the composite baseplate graft complex is then impacted very carefully and gently. Care at this stage is taken to ensure that the 50% rule is followed and the baseplate graft complex has 50% backside coverage of the native scapula. Two lock screws are then inserted, one into the lateral pillar and one into the coracoid pillar, to ensure a satisfactory fixation of the graft. An additional third screw is then inserted as a matter of preference depending on the best area of bone contact, which varies from an anterior to a posterior defect. In cases where the 50% rule is not achieved intraoperatively, a two-stage bone grafting procedure is recommended [7].

##### “Figure 7” Grafts

“Figure 7” grafts can be hand-shaped on the table from the femoral head using the sagittal saw while held with Kocher forceps. Alternatively, they can be pre-cut to size and shaped by the bone bank. The graft is then reamed using a circular reamer and the central hole is created using the peg drill. Then the graft is inserted onto the baseplate. The composite baseplate graft complex is then impacted onto the scapula. This is usually performed over the initial guidewire inserted onto the glenoid, which then allows adequate placement of the bone graft. A supplementary anterior–posterior (AP) screw is not usually needed but can be used based on the surgeon’s preference. If utilised, then it is advised that the graft is fixated onto the glenoid and held with provisional K-wires, and the graft is fixated onto the glenoid neck using the AP screw.

#### 4.2.4. Graft Fixation and Position Considerations

Once the composite baseplate graft complex is well-fixed, a bone hook is used, and the implant is rocked to ensure satisfactory primary fixation is attained intraoperatively. If a satisfactory primary fixation is not attained, the screw in the baseplate construct needs to be removed. The revision and further manoeuvres need to ensure that satisfactory baseplate fixation is achieved. This may require additional impaction bone grafting around the peg or a change to a large central screw-type baseplate.

Utmost care is needed to ensure that the baseplate is positioned with at least a 10° and up to 15° of inferior tilt, and that the screws are adequately positioned in the columns to allow concentric compression and avoid shear forces on the graft. The humeral component is then inserted as per the requirements of the arthroplasty. An attempt is then made to repair the posterior cuff and the subscapularis. If the posterior cuff is deficient, then latissimus dorsi transfer is performed. Care must be taken to avoid over-lateralization of the joint line as the axillary nerve is at risk and at maximal stretch with the glenoid grafting. Palpation of the axillary nerve is performed to confirm there is no tension. In revision cases, a nerve stimulator is routinely used to ensure that the axillary nerve is tension-free and safe. The overall lateralization of the implant can be balanced with the size of the glenosphere based on the morphology and the body habitus of the patient.

#### 4.2.5. Postoperative Protocol

Postoperatively, the shoulder is immobilized using a 60-degree abduction brace for 6 weeks. This position allows compression of the glenosphere, which in turn compresses the graft. This is an essential postoperative step, as it allows concentric loading of the bone graft and avoids eccentric shear forces. Active and passive range of motion are commenced from day one, including wall walks and passive external rotation up to 40°. At 6 weeks postoperatively, the aim of rehabilitation is for the patient to achieve an active assisted elevation of 150° and supine elevation of at least 140°, with active external rotation encouraged to reach 30°. At 10 weeks postoperatively, a routine CT scan is performed to evaluate graft incorporation and stability of the baseplate glenoid construct.

## 5. Preliminary Results

Patients were identified from a single-surgeon single-institution prospectively collected database from August 2016 to December 2022. Patients were eligible for inclusion if they had undergone RSA with glenoid grafting using a structural allograft. Patients were excluded if they had less than 6 months of clinical or radiological follow-up. 

### 5.1. Clinical Follow-Up

Patient-reported and clinical outcomes were completed pre-operatively, at 6 months, 1 year and 2 years by both patients and clinicians, respectively. Patient-reported outcomes (PROMs) included visual analogue score of pain (VAS), American Shoulder and Elbow Surgeons score (ASES), Constant–Murley score (CS), and satisfaction before and after operation. Range of motion (ROM) was assessed by fellowship-trained shoulder surgeons who were not the operating surgeon. ROM included forward flexion, abduction, external rotation in shoulder adduction (ER1), external rotation in shoulder abduction (ER2), internal rotation in shoulder adduction (IR1), and in shoulder abduction (IR2). Both PROMS and ROM were documented in the prospective database (Akunah PROMs, Akunah Medical Technology, Brisbane, Australia). 

### 5.2. Radiographic Follow-Up

**Figure 8:**
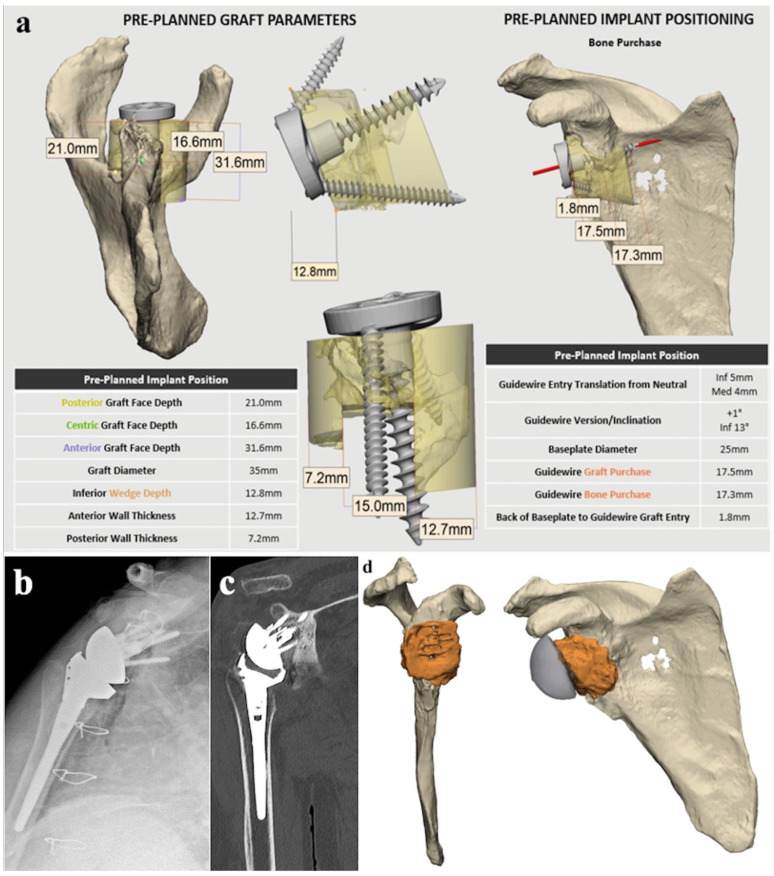
(**a**) Outcome of Akunah preoperative planning, displaying composite graft design (yellow) and measurements. (**b**) Postoperative X-ray and (**c**) 6-month postoperative CT. (**d**) 3D reconstruction of postoperative CT highlighting the graft (orange).

As part of standard clinical practice, CT scans were performed at 10 weeks postoperatively to assess graft healing, 6 months and at 2 years postoperatively to assess any osteolysis and loosening (Figure 8). Assessment was conducted by fellowship-trained shoulder surgeons who were not the operating surgeon. 

### 5.3. Results

There were 28 patients (29 shoulders) (mean age 71, range 57–84) who underwent RSA with glenoid grafting using a structural femoral head allograft. Forty-six percent (46%) were females, while 54% were males (Table 2). In total, 25 out of the 28 patients underwent a successful single-stage procedure. Over half of the cases were revision surgeries (*n* = 16, 55%). Among these, 75% of cases (*n* = 12) were performed in a single-stage revision. The remainder were conducted in two stages (*n* = 4), all of which were revised for infection. Among the primary cases (*n* = 13, 45%), glenoid reconstruction with bone grafting was successfully achieved in one stage in 92% of cases. The only case that required a two-stage reconstruction had a very small vault; hence, the 50% rule could not be met. 

Twenty-one patients were available for follow-up. After a mean follow-up of 16 months (range 6–60), pain, function, and ROM significantly improved compared to preoperative status. VAS improved from five to one (*p* = 0.014). Constant score improved from 22 to 58 (*p* < 0.001). ASES Shoulder score increased from 35 to 73 (*p* < 0.001). All planes of motion significantly improved as well, as seen in Table 3.

The graft healing rate was satisfactory at 92%. Among the patients who had postoperative CT scans available (*n* = 26), only two cases were noted to have had graft resorption with glenoid component loosening at 1 and 2 years postoperatively, of which one of the patients underwent revision surgery using an augmented baseplate. The first patient was on weekly plasmapheresis for cryoglobulinemia, and a graft was required because of an intraoperative glenoid fracture due to poor glenoid stock. This case was performed in 2016, when custom/augmented baseplates were unavailable locally and, an allograft was required intraoperatively to achieve a stable construct. The second patient who had allograft resorption was taking denosumab, which has since been ceased, and is under surveillance with annual radiographs.

Other complications included one case of periprosthetic humeral fracture secondary to a fall at 12 months postoperatively, which required fixation, one case of instability successfully managed with closed reduction, one case of acromial spine fracture that was subsequently repaired, and one patient who developed persistent ulnar nerve symptoms postoperatively. The latter was presumably attributed to postoperative lateralization from a severely medialized glenoid and proximal head migration preoperatively. This results in an overall complication rate of 21%, but with only 8% allograft-related complications. There have been no postoperative infections to date, even in the revision cases with a preoperative infection where an allograft was used for revision reconstruction.

## 6. Discussion

The use of structural femoral allografts for large glenoid defects has been largely successful, with a 92% healing rate in this single-surgeon series when the 50% rule is adhered to and meticulous glenoid bed preparation is performed. The major benefit of using structural allografts in these cases was the ability to restore the joint line and vault stability while maintaining bone stock in case of future revision [7]. Additionally, there was no donor site morbidity, plus the lead-in preparation time and cost of a custom glenoids were avoided, benefitting both the patient and healthcare system costs.

Preoperative planning with metal artifact subtraction software was essential to understand the bony defect and to plan the graft accordingly. Both mirroring and overlaying of the patient’s contralateral scapula onto the pathological glenoid or the generation of an algorithm-based premorbid prediction via SSM were valid methods used to identify the glenoid bone defect [12]. Also, identification of the premorbid joint line via these methods allowed a pre-planned amount of lateralization to be incorporated into the graft size [16]. 

Finally, knowing the size and shape of the defect allowed accurate shaping of the graft either by the bone bank or by hand. This can reduce the graft preparation time in required in the theatre, which can often be vital in large revision cases in which time is of the essence.

When comparing our results to other studies, case series of allografts are of small patient numbers and there are few reported. Radiographic union, graft resorption, baseplate loosening, and infection are among the most important outcomes for allografting. Regarding radiographic follow-up, the most recent and complete series included 20 consecutive patients undergoing femoral head allografts in the primary setting [32]. Their average graft sizes were between 4 mm (2–8 mm) minimum and 15 mm (11–21 mm) maximum, which restored the joint line to a median of 5.7 degrees of retroversion. They reported a graft incorporation rate of 85%, with revision surgery required for the four patients who did not unite, although no further intervention was required in three patients with partial graft resorption. 

A series of 22 patients who underwent RSA with femoral head allografts before July 2016 showed similar graft incorporation rates of 82% at 1 year. This series included 13 revision surgeries, with two patients having radiographic evidence of non-union and one having complete graft resorption. Despite this, revision surgery was declined by all patients, including two who had frank loosening and migration of the baseplate [33]. Of interest, those with baseplate loosening had less correction of the RSA angle than those without baseplate loosening, and two acromial stress fracture non-unions occurred, one in conjunction with baseplate loosening and requiring no further intervention. One further series of both autograft and allograft patients showed high graft complication rates. In the six allograft patients, of which all were revision cases, two of the baseplates failed [34].

Infection following allografting occurred in two patients in the first case series, and allografting may have contributed to graft resorption and baseplate failure in one of the cases [32]. In the second and third series, no cases of infection were noted [32,34]. Our postoperative clinical outcome scores in our complex cases were comparable to the two case series, with an average constant score of 62 [32] and ASES of 70 [33] compared to our 58 and 73, respectively. 

Despite some surgeons’ hesitancy with the use of bone allografts, due to doubts about its healing potential, our series has shown a high rate of graft healing and incorporation. We could attribute this to the compressive forces brought about by the RSA construct and the use of the 60-degree abduction brace, which negates shear forces on the baseplate-bone graft junction and subsequently helps maintain the compressive forces. We also strictly follow the 50% rule to ensure that the construct is stable, and this also facilitates the healing of the bone graft onto the native glenoid.

Although our series showed satisfactory outcomes and graft healing after the use of allografts for glenoid reconstruction, this study’s limitations included a relatively small sample size and mean clinical follow-up of less than 2 years from surgery. A bigger sample size and longer clinical follow-up are necessary to ascertain the long-term outcomes of glenoid grafting using allografts. 

## 7. Conclusions

Varying degrees of glenoid bone defects are commonly encountered in RSA. Achieving successful outcomes requires an individualized approach and decision-making in glenoid reconstruction. The preoperative evaluation and classification of the glenoid defect, along with preoperative planning, guide the selection of the most appropriate glenoid grafting technique. Our findings suggest that structural femoral head allografts are a viable option for large glenoid defects when humeral autografts are unavailable or of poor size and quality. The use of composite grafts in this complex glenoid bone loss series has shown excellent clinical outcomes, with only an 8% graft resorption rate.

## Figures and Tables

**Table 1:** Contraindications for bone graft implantation and harvest.

Contraindications to Bone Graft Implantation	Relative Contraindications to Humeral Autograft Harvest	Absolute Contraindications to Humeral Autograft Harvest
Denosumab	Osteopenia	Tumour
Active infection	Previous cuff repair	Infection
	Proximal humeral fracture	
	Avascular necrosis	

**Table 2:** Demographics of patients who underwent RSA with glenoid grafting using a structural femoral head allograft.

Variable	*n* (%)
Sex	
Male Female	15 (54%) 13 (46%)
Surgery	
Primary One-stage bone grafting procedure Two-stage bone grafting procedure Revision One-stage revision Two-stage revision	13 (45%) 12 (92%) 1 (8%) 16 (55%) 12 (75%) 4 (25%)
Seebauer-Gupta Classification C0 E0 C1 E1 C1 E2 C2 E1 C2 E2 C2 E3 C3 E0 C3 E1 C3 E2 C3 E3 C3 E4 C4 E2	1 (3%) 4 (14%) 2 (7%) 2 (2%) 5 (17%) 2 (7%) 2 (7%) 1 (3%) 1 (3%) 4 (14%) 3 (10%) 2 (7%)
Type of Graft “Figure 7” Wedge	5 (17%) 24 (83%)
Diagnosis Primary Osteoarthritis Cuff tear arthropathy Rheumatoid arthritis Proximal humerus fracture Fracture malunion Irreducible anterior dislocation Revision Infection Implant loosening Failed anatomic TSA Failed hemiarthroplasty Component malposition Pain Instability Periprosthetic fracture	4 (30.8%) 4 (30.8%) 1 (7.7%) 2 (15.4%) 1 (7.7%) 1 (7.7%) 6 (37.5%) 2 (12.5%) 2 (12.5%) 1 (6.3%) 1 (6.3%) 2 (12.5%) 1 (6.3%) 1 (6.3%)

**Table 3:** Clinical and Functional Outcomes.

Parameters	Preoperative	Postoperative	*p* Value
VAS (mean ± SD)	5 ± 4	1 ± 2	0.014
Range of Motion (mean ± SD)			
Forward Flexion (deg) Lateral Elevation (deg) External Rotation (deg) Internal Rotation (deg)	69 ± 39 64 ± 36 7 ± 16 12 ± 15	141 ± 32 122 ± 30 35 ± 20 49 ± 20	<0.001 0.002 <0.001 <0.001
PROMs (mean ± SD) ASES Constant Score	34 ± 23 22 ± 12	73 ± 22 58 ± 14	<0.001 <0.001
Satisfaction (%)	10%	76%	

## Data Availability

Data are contained within the article.

## References

[1] Wagner E., Houdek M.T., Elhassan B.T., Sanchez-Sotelo J., Sperling J.W., Cofield R.H. (2016). Glenoid Bone-Grafting in Revision to a Reverse Total Shoulder Arthroplasty: Surgical Technique. JBJS Essent. Surg. Tech..

[2] Delloye C., Cornu O., Druez V., Barbier O. (2007). Bone allografts. J. Bone Jt. Surg. Br. Vol..

[3] Iannotti J.P., Frangiamore S.J. (2012). Fate of large structural allograft for treatment of severe uncontained glenoid bone deficiency. J. Shoulder Elb. Surg..

[4] Collotte P., Gauci M.-O., Vieira T.D., Walch G. (2022). Bony increased-offset reverse total shoulder arthroplasty (BIO-RSA) associated with an eccentric glenosphere and an onlay 135° humeral component: Clinical and radiological outcomes at a minimum 2-year follow-up. JSES Int..

[5] Walch G., Badet R., Boulahia A., Khoury A. (1999). Morphologic study of the glenoid in primary glenohumeral osteoarthritis. J. Arthroplast..

[6] Bercik M.J., Kruse K., Yalizis M., Gauci M.O., Chaoui J., Walch G. (2016). A modification to the Walch classification of the glenoid in primary glenohumeral osteoarthritis using three-dimensional imaging. J. Shoulder Elb. Surg..

[7] Gupta A., Thussbas C., Koch M., Seebauer L. (2018). Management of glenoid bone defects with reverse shoulder arthroplasty-surgical technique and clinical outcomes. J. Shoulder Elb. Surg..

[8] Boileau P., Moineau G., Roussanne Y., O’Shea K. (2017). Bony Increased Offset-Reversed Shoulder Arthroplasty (BIO-RSA). JBJS Essent. Surg. Tech..

[9] Italia K., Launay M., Gilliland L., Nielsen J., Pareyon R., Hollman F., Salhi A., Maharaj J., Jomaa M., Cutbush K. (2022). Single-Stage Revision Reverse Shoulder Arthroplasty: Preoperative Planning, Surgical Technique, and Mixed Reality Execution. J. Clin. Med..

[10] Mehta N., Nicholson G.P. (2023). Management of Glenoid Bone Loss in Primary Reverse Total Shoulder Arthroplasty. Curr. Rev. Musculoskelet. Med..

[11] Van de Kleut M.L., Yuan X., Teeter M.G., Athwal G.S. (2022). Bony increased-offset reverse shoulder arthroplasty vs. metal augments in reverse shoulder arthroplasty: A prospective, randomized clinical trial with 2-year follow-up. J. Shoulder Elb. Surg..

[12] Gilliland L., Launay M., Salhi A., Green N., Maharaj J., Italia K.R., Cutbush K., Gupta A. (2023). Restoration of glenoid joint line: A three-dimensional analysis of scapular landmarks. JSES Int..

[13] Boileau P., Moineau G., Roussanne Y., O’Shea K. (2011). Bony increased-offset reversed shoulder arthroplasty: Minimizing scapular impingement while maximizing glenoid fixation. Clin. Orthop. Relat. Res..

[14] Cronin K.J., Kirsch J.M., Gates S., Patel M.S., Joyce C.D., Hill B.W., Gutman M.J., Williams G.R., Namdari S. (2021). Three-dimensional measures of posterior bone loss and retroversion in Walch B2 glenoids predict the need for an augmented anatomic glenoid component. J. Shoulder Elb. Surg..

[15] Wagner E.R., Muniz A.R., Chang M.J., Hunt T., Welp K.M., Woodmass J.M., Higgins L., Chen N. (2021). Neuroapraxia and early complications after reverse shoulder arthroplasty with glenoid bone grafting. J. Shoulder Elb. Surg..

[16] Italia K.R., Green N., Maharaj J., Launay M., Gupta A. (2021). Computed tomographic evaluation of glenoid joint line restoration with glenoid bone grafting and reverse shoulder arthroplasty in patients with significant glenoid bone loss. J. Shoulder Elb. Surg..

[17] Smith P.N.G.D., McAuliffe M.J., McDougall C., Stoney J.D., Vertullo C.J., Wall C.J., Corfield S., Page R., Cuthbert A.R., Du P. (2023). . Hip, Knee and Shoulder Arthroplasty: 2023 Annual Report, Australian Orthopaedic Association National Joint Replacement Registry.

[18] Favard L., Lautmann S., Sirveaux F., Oudet D., Kerjean Y., Huguet D., Walch G., Boileau P., Molé D. (2001). Hemiarthroplasty versus reverse arthroplasty in the treatment of osteoarthritis with massive rotator cuff tear. 2000 shoulder prostheses. Two to ten year follow-up.

[19] Tashjian R.Z., Beck L., Stertz I., Chalmers P.N. (2021). Preoperative three-dimensional computer planning for reverse total shoulder arthroplasty and bone grafting for severe glenoid deformity. Shoulder Elb..

[20] Favard L., Berhouet J., Walch G., Chaoui J., Lévigne C. (2017). Superior glenoid inclination and glenoid bone loss: Definition, assessment, biomechanical consequences, and surgical options. Orthopade.

[21] Rachuene P.A., Dey R., Sivarasu S., du Plessis J.P., Roche S., Vrettos B. (2023). A narrative review of treatment strategies for major glenoid defects during primary reverse shoulder arthroplasty, with a focus on the use of structural bone graft. EFORT Open Rev..

[22] Peidro L., Segur J.M., Poggio D., de Retana P.F. (2006). Use of freeze-dried bone allograft with platelet-derived growth factor for revision of a glenoid component. J. Bone Jt. Surg. Br..

[23] Di Felice Ardente P., Fusaro F.M., Abad M.P., Soldado F., Coll J.Q. (2020). The utilization of computer planning and 3D-printed guide in the surgical management of a reverse Hill-Sachs lesion. JSES Int..

[24] Smucny M., Miniaci A. (2018). Pre-shaped Allograft for Glenoid Reconstruction in Anterior Shoulder Instability. Arthrosc. Tech..

[25] Scalise J.J., Iannotti J.P. (2008). Bone grafting severe glenoid defects in revision shoulder arthroplasty. Clin. Orthop. Relat. Res..

[26] Ozgur S.E., Sadeghpour R., Norris T.R. (2017). Revision shoulder arthroplasty with a reverse shoulder prosthesis: Use of structural allograft for glenoid bone loss. Orthopade.

[27] Lopiz Y., García-Fernández C., Arriaza A., Rizo B., Marcelo H., Marco F. (2017). Midterm outcomes of bone grafting in glenoid defects treated with reverse shoulder arthroplasty. J. Shoulder Elb. Surg..

[28] Costain D.J., Crawford R.W. (2009). Fresh-frozen vs. irradiated allograft bone in orthopaedic reconstructive surgery. Injury.

[29] Yu D., Panesar P.S., Delman C., Van B.W., Wilson M.D., Le H.V., Roberto R., Javidan Y., Klineberg E.O. (2022). Comparing Fusion Rates Between Fresh-Frozen and Freeze-Dried Allografts in Anterior Cervical Discectomy and Fusion. World Neurosurg. X.

[30] An H.S., Lynch K., Toth J. (1995). Prospective comparison of autograft vs. allograft for adult posterolateral lumbar spine fusion: Differences among freeze-dried, frozen, and mixed grafts. Clin. Spine Surg..

[31] Wu G., van der Helm F.C., Veeger H.E., Makhsous M., Van Roy P., Anglin C., Nagels J., Karduna A.R., McQuade K., Wang X. (2005). ISB recommendation on definitions of joint coordinate systems of various joints for the reporting of human joint motion--Part II: Shoulder, elbow, wrist and hand. J. Biomech..

[32] Castricini R., Mercurio M., Galasso O., Sanzo V., De Gori M., De Benedetto M., Orlando N., Gasparini G. (2023). Femoral head allograft for glenoid bone loss in primary reverse shoulder arthroplasty: Functional and radiological outcomes. J. Shoulder Elb. Surg..

[33] Tashjian R.Z., Broschinsky K., Stertz I., Chalmers P.N. (2020). Structural glenoid allograft reconstruction during reverse total shoulder arthroplasty. J. Shoulder Elb. Surg..

[34] Ho J.C., Thakar O., Chan W.W., Nicholson T., Williams G.R., Namdari S. (2020). Early radiographic failure of reverse total shoulder arthroplasty with structural bone graft for glenoid bone loss. J. Shoulder Elb. Surg..

